# Preventive effects of *Flos Perariae *(*Gehua*) water extract and its active ingredient puerarin in rodent alcoholism models

**DOI:** 10.1186/1749-8546-5-36

**Published:** 2010-10-26

**Authors:** Zaijun Zhang, Sha Li, Jie Jiang, Pei Yu, Jing Liang, Yuqiang Wang

**Affiliations:** 1 Institute of New Drug Research and Guangdong Province Key Laboratory of Pharmacodynamic Constituents of Traditional Chinese Medicine & New Drug Research, Jinan University College of Pharmacy, Guangzhou 510632, PR China; 2 Division of Oral Biology & Medicine, UCLA School of Dentistry, Los Angeles, CA 90095-1668, USA

## Abstract

**Background:**

*Radix Puerariae *is used in Chinese medicine to treat alcohol addiction and intoxication. The present study investigates the effects of *Flos puerariae *lobatae water extract (FPE) and its active ingredient puerarin on alcoholism using rodent models.

**Methods:**

Alcoholic animals were given FPE or puerarin by oral intubation prior or after alcohol treatment. The loss of righting reflex (LORR) assay was used to evaluate sedative/hypnotic effects. Changes of gama-aminobutyric acid type A receptor (GABA_A_R) subunits induced by alcohol treatment in hippocampus were measured with western blot. In alcoholic mice, body weight gain was monitored throughout the experiments. Alcohol dehydrogenase (ADH) levels in liver were measured.

**Results:**

FPE and puerarin pretreatment significantly prolonged the time of LORR induced by diazepam in acute alcoholic rat. Puerarin increased expression of gama-aminobutyric acid type A receptor alpha1 subunit and decreased expression of alpha4 subunit. In chronic alcoholic mice, puerarin pretreatment significantly increased body weight and liver ADH activity in a dose-dependent manner. Puerarin pretreatment, but not post-treatment, can reverse the changes of gama-aminobutyric acid type A receptor subunit expression and increase ADH activity in alcoholism models.

**Conclusion:**

The present study demonstrates that FPE and its active ingredient puerarin have preventive effects on alcoholism related disorders.

## Background

Alcoholism is a major social, economic and public health problem with profound impacts on brain functions and behaviors [1], exhibiting a variety of symptoms such as hyperexcitability, anxiety, insomnia, agitation and sometimes seizures [2,3]. When alcohol-dependent patients stop drinking, alcohol withdrawal syndromes (AWS) may develop with symptoms of hyperexcitability, anxiety and sleep disorders. The severity of alcohol dependence is positively correlated to the number of intoxication and withdrawal cycles [4]. These clinical findings are supported by studies in rodents [5,6].

Chinese herbal medicines such as *Radix Puerariae *(*Gegen*), *Flos Puerariae *(*Gehua*) and *Hovenia dulcis *(*Zhiju*) and Chinese medicine formulae such as *Gehuajiexing Tang*, *Zhige Yin *and *Wuling San *are used to relieve alcohol hangover [7]. Other natural products such as ginseng, mung bean, rice bean, radish and dandelion are also used as hangover remedies in folk medicine [8].

*Radix Pueraria *belongs to the genus *Pueraria *which includes about 20 species. Keung *et al*. demonstrated that a crude extract of *Radix Puerariae *suppressed ethanol intake of the ethanol-preferring golden Syrian hamsters and identified daidzin and daidzein as the main active components [9]. A population of male and female 'heavy' alcohol drinkers treated with *Radix Pueraria *extract significantly reduced their beer consumption [10]. The underlying mechanism which may be related to both alcohol metabolism and the reward circuits in the brain [11].

Isoflavones including daidzein, daidzin and puerarin are active compounds of *Pueraria*. Daidzin reduced alcohol consumption in laboratory animals [12,13] by raising the monoamine oxidase (MAO)/mitochondrial aldehyde dehydrogenase (ALDH) activity ratio [13]. Puerarin reduced voluntary alcohol intake and alcohol withdrawal symptoms in alcohol preferring (P) rats [14]. However, the effects of puerarin on central nervous system and liver metabolism are not clearly understood.

GABA_A_R and ADH are important pharmacological concerns in alcoholism [15,16]. The changes in levels of several GABA_A_R subunits [17] caused by alcohol are accompanied by behavioral disorders, e.g., loss of righting reflex (LORR) [17-19]. The primary pathway of alcohol metabolism involves oxidation to acetaldehyde, catalyzed by ADH, and followed by further oxidation to acetate, catalyzed by ALDH [20]. Therefore, ADH is one of the most important enzymes for decreasing alcohol concentration in the body.

The present study investigates the preventive effects of *Flos Puerariae *extract (FPE) and its main active component puerarin in acute and chronic alcohol intoxicated animals.

## Methods

### FPE preparation

*Flos Puerariae *was purchased from a local Chinese medicine shop and authenticated by an investigator (JJ) in pharmaceutical botany. The authentication procedure included appearance identification of raw material and comparison of chemical constituents which have described in Zhong-Yao-Zhi [21]. A voucher herbarium specimen of the material used in this study was deposited as specimen No.125 in the Herbarium of the College of Pharmacy, Jinan University (PR China). The crude herb (300 g) was boiled for two hours at 100°C in 1500 ml distilled water. The supernatant was collected after centrifugation and concentrated to 1 g/ml. Fourteen (14) chemical standards, namely 4'-*O*-glucopyranoside, 3'-methyoxy-4'-*O*-glucopyranoside, 4',7-*O*-glucopyranoside, puerarin, 6'-*O*- xylosylpuerarin, mirificin, daidzin, 3'-methoxypuerarin, genistin, sophoraside A, ononin, daidzein, genistein and formononetin, were purchased from the National Institute for the Control of Pharmaceutical and Biological Products, Beijing, PR China.

### Quality control of FPE

The qualitative analysis of FPE was performed on an Agilent 1200 Series Reverse-Phase Liquid Chromatography (RPLC) system (Agilent Technologies, Germany) equipped with a microvacuum degasser, a high pressure binary pump, an autosampler, a column compartment coupled with a carrier for heat exchanger (1.6 μl), a diode array detector connected to Masshunter software (A02.02, Agilent Technologies, Germany). A Zorbax SB C18 column (4.6 mm×50 mm, 1.8 μm, Agilent Technologies, Germany) was used. The mobile phase consisted of A (0.1% formic acid) and B (methanol) with gradient elution: 0-3 minutes, 20-30% B; 3-4 minutes, 30-32% B; 4-8 minutes, 32-57% B. Flow rate was 2.0 ml per minute and the injection volume was 4 μl. The column temperature was set at 46°C. Peaks were detected at 250 nm.

### Animals

Male Sprague-Dawley rats (body weight 300-350 g) and male *BALB/C *mice (body weight 30-35 g) were obtained from the Experimental Animal Center of Guangdong Province, China (SPF grade, Certificate No. 2005A047, 2006A059). Rats in acute alcoholic experiments were divided into five groups of six (6) animals per group. Chronic alcoholic mice were also divided into five groups of eight (8) animals per group. All animals were kept on a 12 hour/12 hour light/dark cycle under controlled temperature and humidity with *ad lib *access to food and water. The animal experiments were approved by the Animal Research Ethics Committee, Jinan University.

### Acute alcoholic rat model

A dose of 25% (v/v) alcohol was given by intragastric administration, 2 ml per 100 g body weight, 30 minutes before or after drug treatment. FPE or puerarin was given 1 ml/100 g or 500 mg per kg body weight respectively. After two days of withdrawal, Loss of Righting Reflex (LORR) assay was used to assess the drug's protective effects. Rats were then sacrificed and their hippocampi were dissected for GABA_A_R subunit analysis using western blot.

### Chronic alcoholic mouse model

Alcohol (25%, v/v) was given by intragastric administration, 0.2 ml per 10 g body weight, 30 minutes before or after puerarin treatment 250 mg and 500 mg per kg body weight once a day for 12 days. Body weight of mice was monitored every two days. At the end of experiment, mice were sacrificed and their livers were dissected for Alcohol dehydrogenase (ADH) assay.

### Diazepam-induced LORR assay

Two days after alcohol intoxication and withdrawals, all animals received an intraperitoneal injection of diazepam (30 mg per kg body weight). LORR and recovery of the righting reflex were observed. After each injection, animals were placed in a supine position in a cage with wire lids. LORR was recorded as the time at which the animal was unable to turn itself. Animals were left in the supine position until recovery of the righting reflex. Recovery of the righting reflex was defined as the time that elapsed until the animal was able to right itself three times in 60 seconds. The time to regain the righting reflex was recorded for each animal.

### Protein sample preparation and western blot analysis

After the LORR test, rats were anesthetized and tissues were separated. Individual hippocampi were dissected on ice from each rat brain. P2 membrane fractions were prepared by homogenization, low-speed centrifugation in 0.32 M sucrose and then centrifugation (×12,000 *g*, Beckman J2-21 centrifuge, Beckman Instruments, Germany) of the supernatant for 20 minutes. The pellet was resuspended and washed in 20 volumes of phosphate-buffered saline (PBS, 150 mM NaCl, 10 mM Na_2_HPO_4_/NaH_2_PO_4_, pH7.4). The final pellet was resuspended in five volumes of PBS and protein concentration was determined with Bradford assay kit (Bio-Rad Laboratories, USA).

Aliquots of 40 μg of protein from each sample were separated on 10% SDS-polyacrylamide gel electrophoresis. Then the proteins were transferred to polyvinylidene difluoride membranes. Blots were stained with anti-peptide α1 or α4 antibodies (1:1000, Santa Cruz Biotechnology, USA) followed by horseradish peroxidase-conjugated anti-rabbit antibodies (1:2000, Zymed laboratories, USA) or anti-goat IgG (1:500, Vector laboratories, Canada). Bands were detected by DAB staining (Sigma, USA). Beta-actin antibody (1:1000, Santa Cruz Biotechnology, USA) was used to detect endogenous standard for normalization. The bands from various groups corresponding to the appropriate molecular weight for each subunit were analyzed and values were compared using densitometric measurements with image analysis system.

### ADH assay

At the end of chronic alcoholic treatment, mice were sacrificed and livers were dissected on ice. Liver homogenates were prepared with manual homogenization in a 2 mL glass pestal and centrifugation (×3000 *g*, Beckman J2-21 centrifuge, Beckman Instruments, Germany) for 10 minutes. Supernatants were collected for ADH determination. ADH assay kit was purchased from Nanjing Jiancheng Biological Laboratory (China) and the experiment was performed according to the manufacturer's instructions. Briefly, oxidized form of nicotinamide-adenine dinucleotide (NAD) was added to the liver sample. The absorbance of the reaction mixture was recorded at 340 nm, and ADH activity was calculated from the absorbance value and protein content. ADH activity was expressed in unit per mg protein (U/mg), i.e. 1 U/mg means that ADH yields 1 nmol product with 1 mg protein per minute at 37°C.

### Statistical analysis

Data were expressed as mean ± SD for the number (*n*) of animals in each group. ANOVA and Tukey post-test were performed to determine the significant differences between various groups using GraphPad Prism 5.0 software (GraphPad Software, USA). *P*-values of < 0.05 were considered statistically significant.

## Results

**Figure 1:**
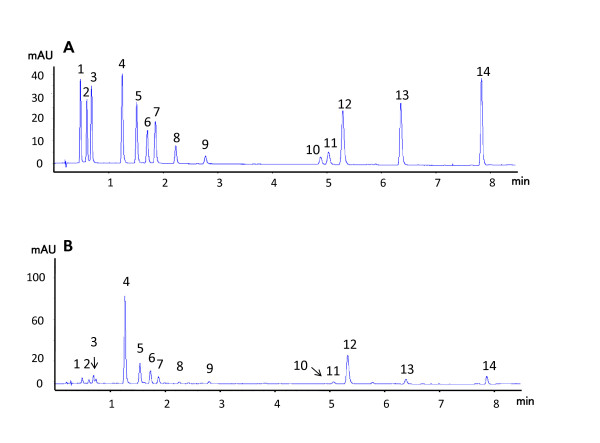
**Typical RRLC chromatograms of mixed standards (A) and FPE (B)**. (1) 4'-*O*-glucopyranoside, (2) 3'-methyoxy-4'-*O*-glucopyranoside, (3) 4',7-*O*-glucopyranoside, (4) puerarin, (5) 6''-*O*- xylosylpuerarin, (6) mirificin, (7) daidzin, (8) 3'-methoxypuerarin, (9) genistin, (10) sophoraside A, (11) ononin, (12) daidzein, (13) genistein, (14) formononetin.

Figure 1 shows the RPLC chromatograms of some standards including puerarin in standard solution and in FPE. Fourteen (14) constituents were identified with RPLC fingerprinting as 4'-*O*-glucopyranoside, 3'-methyoxy-4'-*O*-glucopyranoside, 4',7-*O*-glucopyranoside, puerarin, 6'-*O*- xylosylpuerarin, mirificin, daidzin, 3'-methoxypuerarin, genistin, sophoraside A, ononin, daidzein, genistein and formononetin. Among them, the abundance of puerarin was highest. This RPLC fingerprinting system can be employed as a tool for FPE quality assurance.

**Figure 2:**
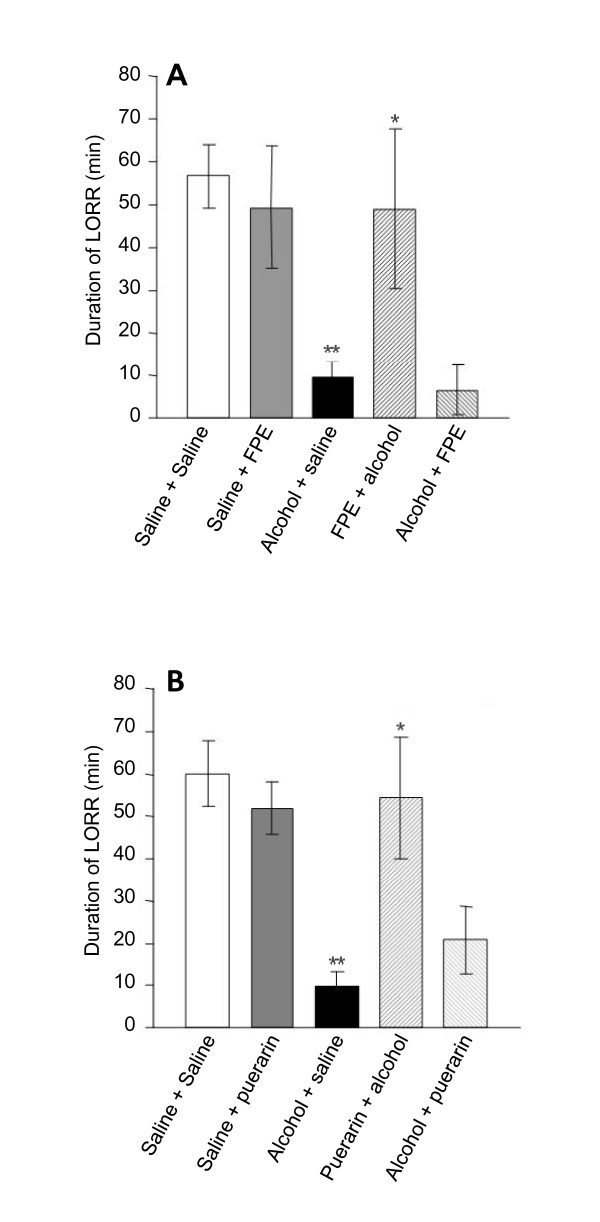
**Effects of FPE (A) and puerarin (B) on duration of diazepam induced LORR in normal and alcoholic rats**. Data are expressed as mean ± SD (*n *= 6). *P < 0.05 compared with 'alcohol+saline' treated group; **P < 0.01 compared with 'saline+saline' treated group.

As shown in Figure 2, LORR induced by diazepam significantly decreased [*P *= 0.0003] in acute alcoholic rats (alcohol + saline group). The duration of diazepam-induced LORR was about 60 minutes in normal rats (saline + saline group, Figure 2); however, LORR of the acute alcoholic rats was at 9.8 ± 3.27 minutes (Figure 2) which was significantly different from that of the normal rats [*P *= 0.0003]. FPE and puerarin alone had no significant effect in the duration of LORR in normal rats. Pretreatment with FPE or puerarin significantly recovered the LORR time of the acute alcoholic rats, which went up to 49 ± 18.64 and 51.83 ± 6.11 minutes respectively, [*P *= 0.001] against alcohol + saline group). However, FPE or puerarin administration post-alcohol treatment did not significantly recover the duration of LORR (Figure 2).

**Figure 3:**
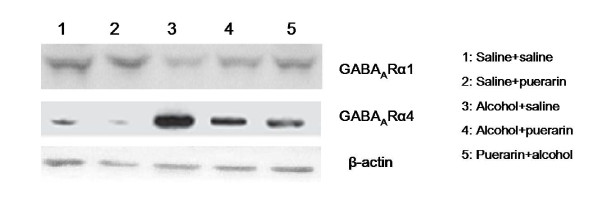
**Representative western blot of protein expression of GABA**_**A**_**R α1 and α4 subunit**.

**Figure 4:**
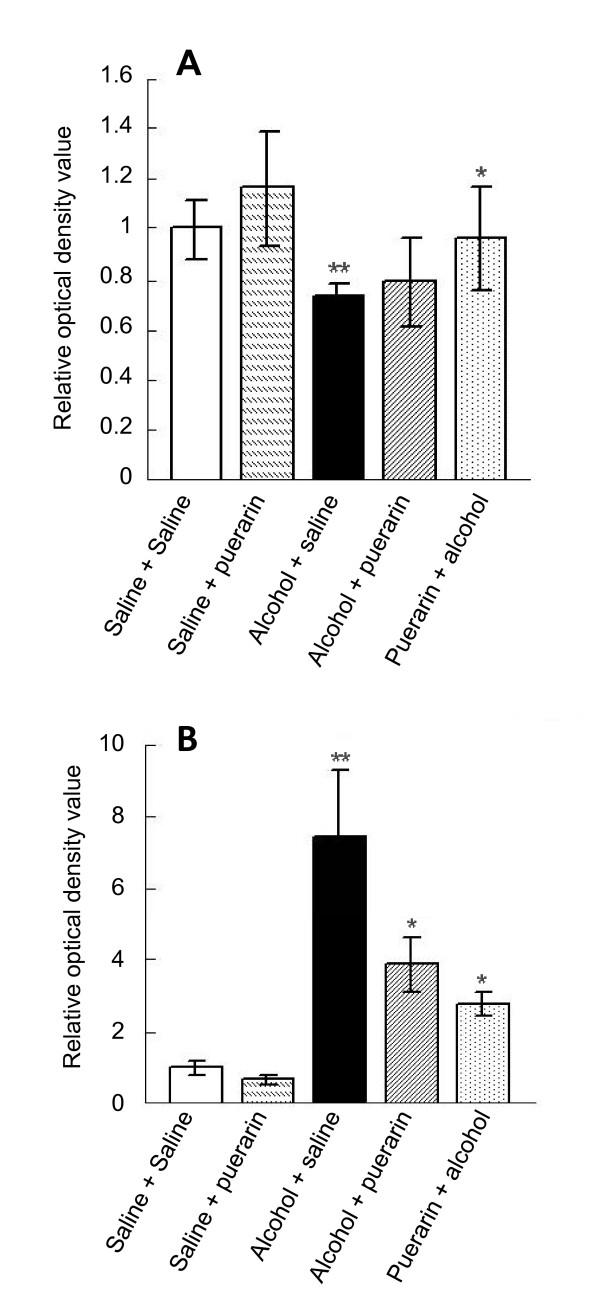
**Effects of puerarin on expression changes of GABA**_**A**_**R α1 and α4 subunits in an alcoholic rat model**. (A) Changes of expression of GABA_A_Rα1; (B) changes of α4 subunit Values are expressed as mean ± SD (*n *= 3). *P < 0.05 compared with 'alcohol+saline' treated group; **P < 0.01 compared with 'saline+saline' treated group.

Alcohol intoxication significantly decreased GABA_A_R α1 subunit expression in the hippocampus (Figure 3 and Figure 4A) whereas GABA_A_R α4 subunit expression was notably increased (Figures 3 and 4B). These results were consistent with those reported previously by Cagetti *et al *[17]. Puerarin pretreatment reversed the effects on GABA_A_R subunit expression changes in alcoholic rats. Puerarin treatment after alcohol administration showed less effect than the puerarin pretreatment.

Alcohol exposure significantly changed weight gain. Specifically, average weight of saline + saline group increased from 20.7 ± 1.25 g to 30.36 ± 2.06 g while that of alcohol + saline group decreased from 22.52 ± 0.43 g to 18 ± 2.88 g [*P *= 0.007] (Table 1). Animals of puerarin + alcohol group weighed significantly more than those of the alcohol + saline group from day 4 to 12 [P = 0.02] (Table 1). Puerarin pretreatment prevented body weight loss in alcoholic mice in a dose-dependent manner.

**Table 1:** Puerarin prevents the loss of body weight in alcoholic mice

	Body weight after days of treatment (g)						
Groups	0 day	2 days	4 days	6 days	8 days	10 days	12 days
**Saline + saline**	20.7 ± 1.25	22.07 ± 0.45	23.87 ± 1.03	25.67 ± 1.36	27.73 ± 1.86	28.10 ± 1.37	30.37 ± 2.06
**Alcohol + saline**	22.52 ± 0.43	15.18 ± 2.05**	12.30 ± 1.35**	15.4 ± 0.29**	16.1 ± 3.1**	16.7 ± 1.33**	18 ± 2.88**
**Puerarin (250) + alcohol**	22.58 ± 1.96	18.23 ± 2.49	17 ± 4.93	18.27 ± 2.35	19.77 ± 3.67	19.43 ± 4.39	20.83 ± 5.15*
**Puerarin (500) + alcohol**	24.22 ± 0.56	19.05 ± 4.73	21.68 ± 2.22*	23.48 ± 2.27*	24.08 ± 1.89*	23.9 ± 2.44*	25.23 ± 1.4*
**Alcohol + puerarin (500)**	23.05 ± 0.44	19.73 ± 3.22	19.53 ± 5.08	20.55 ± 6.3	21.2 ± 7.05	22.23 ± 3.48*	24.77 ± 2.8*

Data are expressed as mean ± SD (n = 8).*P < 0.05 compared with 'alcohol+saline' treated group; **P < 0.05 compared with 'saline+saline' treated group.

**Figure 5:**
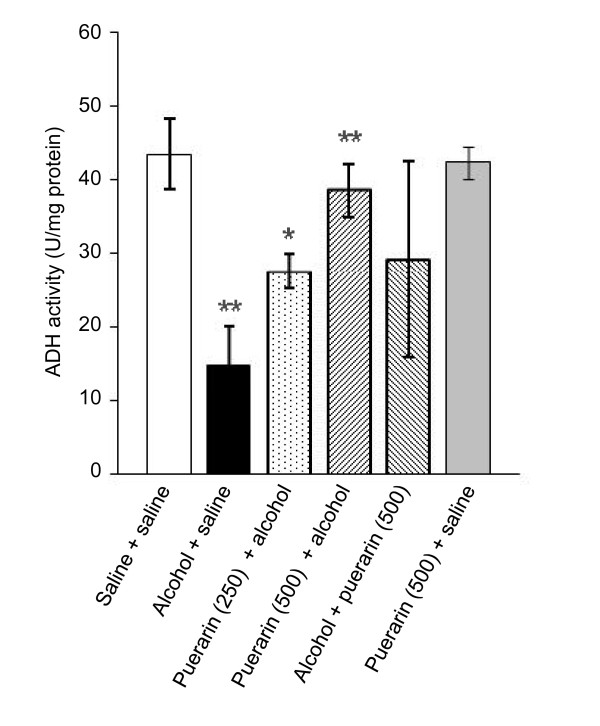
**Puerarin increases the ADH activity in alcoholic mice**. Data are expressed as mean ± SD (*n *= 8). *P < 0.05 compared with 'alcohol+saline' treated group; **P < 0.01 compared with 'saline+saline' treated group.

ADH activity in the alcoholic mice significantly decreased compared to that in normal mice [*P *= 0.002]. Puerarin pretreatment reversed this decrease in ADH activity in the livers of alcoholic mice in a dose-dependent manner (Figure 5), suggesting that puerarin may exert its preventive effects in alcoholism through enhancing ADH activity.

## Discussion

Our results demonstrated that pretreatment of FPE or puerarin had significant anti-anxiety effects in the diazepam-induced LORR assay. However, administration of FPE or puerarin after alcohol treatment had less effect. These data suggest that FPE may prevent but not relieve alcoholic disorders.

GABA_A_Rs are the major targets for actions of alcohol [5,22]. Our previous studies demonstrated that single dose ethanol intoxication leads to GABA_A_R plasticity changes such as transcriptionally increases in α4 and α2 and decreases in α1 subunits with preferential insertion of the newly formed α4βγ2 GABA_A_R at synapses [18,19,23]. To study human alcohol withdrawal and dependence, we established a model for the chronic intermittent ethanol (CIE) intoxication in rats. CIE rats revealed alterations in GABA_A_R subunit composition and subcellular localization [18,23,24]. The present study found that alcohol altered GABA_A_R composition in an acute alcoholic model, i.e. single dose alcohol treatment increased the expression of GABA_A_R α4 subunit and decreased the GABA_A_R α1 subunit. To investigate whether puerarin's LORR recovery effect was due to changes of GABA_A_R subunit, we determined expression of GABA_A_R subunits α1 and α4 using western blot. Puerarin pretreatment reversed these changes significantly, that is, upregulated α1 subunit expression and downregulated α4 subunit expression. However, puerarin post-treatment after alcohol was less effective than puerarin pretreatment in reversing transcriptional changes of GABA_A_R subunits. These data were consistent with puerarin's effect on diazepam-induced LORR recovery.

ADH, which decreases alcohol concentration in the body [20], is one of the most important enzymes in alcohol metabolism. The alcohol concentration in blood increases when ADH activity is decreased, aggravating alcoholic damage to brain, liver and other important organs. Puerarin elevates ADH activity and prevents body weight loss after chronic alcohol exposure. The increase in ADH activity may account for puerarin's detoxification effects against alcohol in liver hepatocytes [25,26]. Apart from the detoxification effects, three isoflavonoid compounds, namely puerarin, daidzin and daidzein isolated from *Pueraria lobata*, suppressed voluntary alcohol consumption in alcohol-preferring rats [27]. It was postulated that the suppression of alcohol reinforcement produced by these compounds is mediated centrally in the brain reward pathway [27,28].

Previous *in vitro *studies showed that daidzin and daidzein, two isoflavonoids structurely similar to puerarin, were potent inhibitors for mitochondrial low-Km aldehyde dehydrogenase and alcohol dehydrogenase separately [29,30]. Therefore, it was postulated at first that these isoflavones might deter alcohol drinking by interfering with alcohol metabolism. However, *in vivo *study showed that neither blood ethanol nor acetaldehyde concentrations were affected in hamsters injected with daidzein [27,31]. These conflicting results warrant further investigations.

Recently, research has focused on the effects of oxidative stress in diseases caused by alcohol [32-36]. When an organism suffered from the stimulation of an oxidant such as alcohol, a large amount of reactive oxygen species (ROS) with neuronal toxicity would be produced and the lipid peroxidation of surrounding tissues increased [37,38]. Cao *et al*. reported that isoflavones and *Pueraria *extracts containing daidzein, daidzin and puerarin had strong anti-oxidative activities [39]. Anti-oxidation may be another mechanism underlying the anti-alcoholism activity of puerarin. Further investigations are warranted.

## Conclusion

The present study demonstrates that FPE and its active ingredient puerarin have preventive effects on alcoholism related disorders. Puerarin pretreatment, but not post-treatment can reverse the changes of GABA_A_R subunit expression and increase ADH activity in alcoholism models.

## Abbreviations

FPE: *Flos puerariae *lobatae water extract; LORR: loss of righting reflex; ADH: Alcohol dehydrogenase; ALDH: aldehyde dehydrogenase; AWS: alcohol withdrawal syndromes; RPLC: Reverse-Phase Liquid Chromatography; PBS: phosphate-buffered saline; GABA_A_R: gama-aminobutyric acid type A receptor;

## Competing interests

The authors declare that they have no competing interests.

## Authors' contributions

ZJZ, SL and JJ carried out the experiments and data analysis. ZJZ and JL interpreted the data and wrote the manuscript. YQW and PY designed the study. All authors read and approved the final version of the manuscript.
